# Analysis of reliability, accuracy, sensitivity and predictive value of a subjective method to classify facial pattern in adults

**DOI:** 10.1590/2177-6709.21.6.058-066.oar

**Published:** 2016

**Authors:** Gilberto Vilanova Queiroz, José Rino, João Batista de Paiva, Leopoldino Capelozza

**Affiliations:** 1 PhD in Orthodontics, University of São Paulo (FOUSP), São Paulo, Brazil.; 2 Associate professor, Department of Orthodontics, University of São Paulo (FOUSP), São Paulo, Brazil.; 3 Professor and chair, Department of Orthodontics, University of São Paulo (FOUSP), São Paulo, Brazil.; 4 Assistant professor, Department of Orthodontics, University of Sagrado Coração, Bauru, São Paulo, Brazil.

**Keywords:** Orthodontics, Diagnosis, Face

## Abstract

**Introduction:**

Craniofacial pattern diagnosis is vital in Orthodontics, as it influences decision-making regarding treatment options and prognosis. Capelozza Filho proposed a subjective method for facial classification comprising five patterns: I, II, III, Long Face and Short Face.

**Objective:**

To investigate the accuracy of a subjective classification method of facial patterns applied to adults.

**Methods:**

A sample consisting of 52 adults was used for this study. Frontal and lateral view photographs were taken with subjects at rest position, including frontal smile. Lateral cephalometric radiographs were organized in a PowerPoint^®^ presentation and submitted to 20 raters. Method performance was assessed by examining reproducibility with Kappa test and calculating accuracy, sensitivity and positive predictive values, for which 70% was set as critical value. The gold standard of the classification was personally set by the author of the method.

**Results:**

Reproducibility was considered moderate (Kappa = 0.501); while accuracy, sensitivity and positive predictive values yielded similar results, but below 70%.

**Conclusions:**

The subjective method of facial classification employed in the present study still needs to have its morphological criteria improved in order to be used to discriminate the five facial patterns.

## RESUMO

**Introdução:**

o diagnóstico do padrão craniofacial é fundamental em Ortodontia, visto que influencia tanto a decisão terapêutica quanto seu prognóstico. Capelozza Filho propôs um método subjetivo de classificação facial em cinco padrões: Padrão I, Padrão II, Padrão III, Face Longa e Face Curta.

**Objetivo:**

investigar o desempenho do método subjetivo de classificação do padrão facial, quando aplicado em adultos.

**Métodos:**

a amostra foi composta por 52 adultos de ambos os sexos. Fotografias frontal e lateral em repouso, frontal em sorriso e telerradiografia em norma lateral foram montadas em apresentação do PowerPoint^®^ e levadas pessoalmente aos vinte examinadores. Para se avaliar o desempenho do método, foi analisada a reprodutibilidade por meio do teste Kappa e calculados os valores de acurácia, sensibilidade e valor preditivo positivo, para os quais adotou-se 70% como valor crítico. O padrão-ouro foi constituído pela classificação realizada pelo autor do método.

**Resultados:**

a reprodutibilidade foi considerada regular (Kappa 0,501) enquanto a acurácia, sensibilidade e valor preditivo positivo obtiveram resultados próximos; porém, abaixo de 70%.

**Conclusão:**

o método subjetivo de classificação facial precisa de aprimoramentos nos critérios morfológicos utilizados para discriminar os cinco padrões faciais.

## INTRODUCTION

Craniofacial pattern description is relevant in orthodontic diagnostics, given that anatomical variations are related to malocclusion severity.^1^ Individuals with similar skeletal architectures grow and respond similarly to orthodontic treatment.^2^ For this reason, clinical studies seeking to establish the effects of dentofacial orthopedics should include growth expectations based on facial typology of both the treated group and the control group.^1^ Selection of samples according to Angle’s occlusal classification (Classes I, II and III) does not ensure the structural homogeneity of groups, since different maxillomandibular relationships that predispose patients to protrusion and retrusion coexist with similar occlusal patterns.^3^ ^,^ ^4^

Given that similar malocclusions can pose different challenges due to facial architecture,^1^ establishing a differential diagnosis of each facial pattern is paramount. Capelozza Filho^5^ organized a diagnostic system that groups faces in five different patterns: Pattern I, featuring skeletal balance; Patterns II and III, characterized by positive and negative sagittal steps between the jaws, respectively; Long Face pattern, exhibiting excessive facial lower third without lip seal;^6^ and Short Face pattern, featuring a deficient facial lower third with forced lip seal. Pattern I involves solely a dental problem, whereas in the other patterns the face and dentoalveolar processes reflect underlying skeletal imbalances.

New diagnostic methods should be incorporated into the medical or dental routine after investigating the accuracy, as well as the success scores when compared to the gold standard.^7^ Assuming that the classification of facial patterns proposed by Capelozza Filho^5^ ^,^ ^6^ is a new diagnostic system, it was considered appropriate to investigate whether such method ensures proper reproducibility and high success scores in the diagnosis of facial patterns in adults.

## MATERIAL AND METHODS

This project was approved by the Ethics Committee board of the School of Dentistry of *Universidade de São Paulo*, registered under protocol #118/2008. The research used 52 Brazilian adults of both genders, of white, black or mixed ethnicity, undergoing orthodontic treatment in private practice or in the graduate course in Orthodontics, School of Dentistry, *Universidade de São Paulo*. Initially, 120 individuals with no history of maxillofacial trauma or surgery were selected and classified by an experienced orthodontist into five facial patterns: I, II, III, Long Face or Short Face. In an effort to avoid compromising the accuracy of the investigation due to rater fatigue, the sample was reduced to 52 individuals, which required random selection by lot in the initial sample of facial patterns I and II.

**Figure 1:**
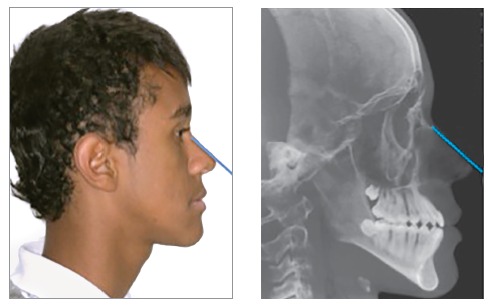
Reference used for obtaining similar inclinations between the horizontal planes of the lateral photograph and radiograph: nasal dorsum.

Frontal and profile photographs were taken with subjects at rest position, smiling in frontal view and lateral view, in addition to cephalometric radiographs. Head positioning in each photograph was verified by the orthodontist responsible for the orthodontic treatment, photographs with inappropriate head position were excluded. Lateral cephalometric radiographs were digitally rotated, so as to obtain inclinations that were similar to those seen in the photographs. Rotation was performed visually by adopting the line of the nasal dorsum relative to the vertical line represented by the right edge of the photographic or radiographic images as reference (Fig 1).

Twenty professionals were invited to carry out the analysis of diagnostic agreement after having been trained on facial pattern classification by Capelozza Filho. The evaluations were gathered into three groups:

I) Experienced professionals: Orthodontics professors who learned the method more than eight years ago (n = 10).
II) Inexperienced professionals: current students of the specialization course coordinated by Capelozza Filho (n = 10).
III) Sum of all professionals, experienced and inexperienced (n = 20).

### Facial pattern classification

**Figure 2:**
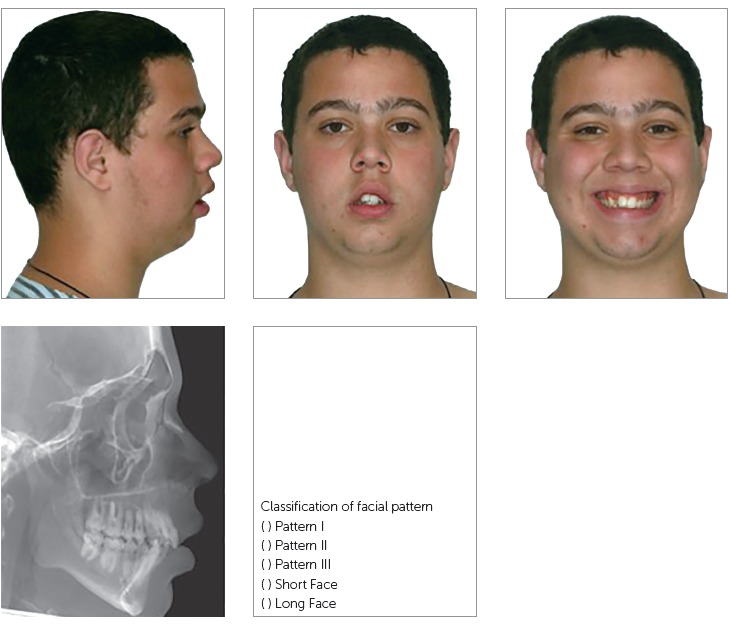
Screen models for assessing the sample.

The photographs and radiographs were imported into a PowerPoint^®^ presentation and personally delivered by the author of this research to the gold standard, represented by Capelozza Filho, and to the 20 raters, who marked one of the following options in each screen: Pattern I, Pattern II, Pattern III, Long Face Pattern or Short Face Pattern (Fig 2).

In order to investigate to what extent agreement exceeded the chance factor, Kappa coefficient^8^ was applied according to the interpretation in Table 1.

**Table 1:** Kappa agreement scale.

Kappa values	Interpretation (strengths)
< 0	Poor agreement
0 - 0.20	Slight agreement
0.21 - 0.40	Fair agreement
0.41 - 0.60	Moderate agreement
0.61 - 0.80	Substantial agreement
0.81 - 0.99	Almost perfect agreement
1	Perfect agreement

Additionally, were calculated the accuracy and operational characteristics, consisting of sensitivity, specificity, false positive, false negative, positive likelihood ratio, positive predictive value and positive post-test probability for estimated clinical prevalence. 

The operational characteristics of the method were determined based on contingency tables as exemplified in Table 2.

**Table 2:** Model contingency table used to determine the operational characteristics of the subjective method of classification of facial patterns.

	Pattern X	Other patterns
Pattern X	TP	FP
	(True positive)	(False positive)
Other patterns	FN	TN
	(False negative)	(True negative)

Reproducibility and accuracy were calculated in the three groups. The gold standard was established by means of the classification made by the creator of the method.

## RESULTS

Kappa results can be found in Table 3. In general, inter-rater agreement was moderate and the strength of agreement between experienced and inexperienced raters was similar. In Table 4, the classification of facial patterns devised by Capelozza Filho, considered the gold standard. The distribution of agreements and disagreements between raters and the gold standard in classifying the facial patterns is shown in Table 5. Of the total 1040 planned evaluations (52 subjects multiplied by 20 raters), four were discarded because the quality of the image, as seen on the computer screen, was considered unsatisfactory. In considering the group comprised of all raters, there were 651 agreements and 385 disagreements. In Table 6, it can be observed that the overall success rate was 62,8%; the experienced group’s accuracy was 66.4%, while the inexperienced group’s accuracy was 58.2%. The operational characteristics of the method for each facial pattern are shown in Table 7. Confounding factors in each pattern can be seen in Table 8. In Patterns II and III, approximately half of the confounders were related to vertical patterns and the other half was related to Pattern I; in the Short and Long Face patterns, 100% of discrepancies involved confounding variables with sagittal patterns.

**Table 3:** Rater’s Kappa index in classifying facial patterns in adults

	Experienced Raters (Group I)		Inexperienced Raters (Group II)		All Raters (Group III)	
	kappa	agreement	kappa	agreement	kappa	agreement
Total sample	0.50*	Moderate	0.50*	Moderate	0.50*	Moderate
Pattern I	0.39*	Fair	0.37*	Fair	0.38*	Fair
Pattern II	0.49*	Moderate	0.54*	Moderate	0.52*	Moderate
Pattern III	0.61*	Substantial	0.52*	Moderate	0.55*	Moderate
Long Face	0.44*	Moderate	0.46*	Moderate	0.46*	Moderate
Short Face	0.64*	Substantial	0.69*	Substantial	0.64*	Substantial

Kappa of the category p < 0.01.

**Table 4:** Frequency of facial patterns classified by the gold standard.

Patterns	Pattern I	Pattern II	Pattern III	Long Face	Short Face
Frequency	14	15	11	9	3

**Table 5:** Agreements (in bold) and disagreements between raters and the gold standard.

OVERALL	Pattern I	Pattern II	Pattern III	Long Face	Short Face	Rater results
Pattern I	165	59	42	21	0	287
Pattern II	25	179	1	16	3	224
Pattern III	40	0	141	32	2	215
Long Face	9	21	21	111	0	162
Short Face	41	38	14	0	55	148
Gold standard results x 20	280	297	219	180	60	1036

**Table 6:** Overall success rates (all raters), and separate success rates for the group of experienced and inexperienced raters.

	Pattern I	Pattern II	Pattern III	Long Face	Short Face	Success scores	Total ratings	Accuracy
All raters	165	179	141	111	55	651	1036	62.83%
Experienced raters	84	95	82	54	28	343	516	66.4%
Inexperienced raters	81	84	59	57	27	308	520	58.2%

**Table 7:** Calculation of operational characteristics and post-test probability for facial pattern classification.

OVERALL	Pattern I	Pattern II	Pattern III	Long Face	Short Face
Sensitivity	58.93%	60.27%	64.38%	61.67%	91.67%
False negatives	41.07%	39.73%	35.62%	38.33%	8.33%
Specificity	83.86%	93.91%	90.94%	94.04%	90.47%
False positives	16.14%	6.09%	9.06%	5.96%	9.53%
Positive likehood ratio	3.65	9.9	7.11	10.35	9.62
Prevalence in the sample	27.03%	28.67%	21.14%	17.37%	5.79%
Positive Predictive Value	57.49%	79.91%	65.58%	68.52%	37.16%
Estimated clinical prevalence	40%	32%	8%	15%	5%
Post-test probability	70.88%	82.32%	38.19%	64.61%	33.61%

**Table 8:** Success rates (in bold) and confounders in each facial pattern.

Facial patterns	Pattern I	Pattern II	Pattern III	Long Face	Short Face
Pattern I	59%	20%	20%	12%	0%
Pattern II	9%	60%	0%	9%	5%
Pattern III	14%	0%	64%	18%	3%
Long Face	3%	7%	10%	61%	0%
Short Face	15%	13%	6%	0%	92%
Overall	100%	100%	100%	100%	100%

## DISCUSSION

The diagnosis of maxillomandibular relationships requires objective criteria and precise language for high agreement among professionals.^9^ ^,^ ^10^ The evaluation method of facial pattern proposed by Capelozza Filho^5^ ^,^ ^6^ have such requirements, but its performance has not yet been evaluated. The objective of this study was to analyze the performance of this method to classify a sample consisting of 52 adults of both genders.

Reis et al^11^ e Vaz et al^12^ evaluated intra- and inter-rater reproducibility among experienced orthodontists. Intra-rater reproducibility, in all studies, was adequate, which proved the efficiency of the method in this particular aspect. Moreover, inter-operator reproducibility was just moderate. One possible explanation for this moderate Kappa value might be related to the fact that raters had no access to the lateral cephalometric radiographs when classifying the facial patterns, which restricted the analysis of dental and skeletal morphology. This study selected 10 experienced raters from the 16 used in the study by Reis et al,^11^ and employed both facial images and lateral view cephalometric radiographs. However, the reproducibility of experienced raters also showed moderate Kappa index values, which indicated the important role of the soft tissue criterion in classifying facial pattern and little influence of lateral radiograph in orthodontic diagnosis, which is in accordance with Durão et al.^13^

To investigate whether professional experience influences method reproducibility, Kappa coefficient results were calculated separately for the groups of experienced and inexperienced raters (Table 3). Strength of agreement between the two groups of raters was similar, except for Pattern III, which shows that, in general, professional experience time exerted no influence on method reproducibility.

Inter-rater agreement does not always reflect a truthful diagnosis; therefore, having identical diagnoses does not imply correctness. In order to investigate the method’s success rate, it is necessary to compare its results to a gold standard. This study used the classification of facial patterns devised by the author of the method as the gold standard. However, this does not mean that classification represents the absolute gold standard. In fact, it was determined that the author’s results are the gold standard, but only for professionals who use his method. It is worth noting that in the absence of an absolute gold standard for comparing the results obtained by this subjective diagnostic method, this investigation cannot be considered a diagnostic method validation research.

The gold standard used to classify the sample results is shown in Table 4, whereas the distribution of agreements and disagreements between raters and the gold standard in classifying the facial patterns is shown in Table 5. Of the total 1040 planned evaluations (52 subjects multiplied by 20 raters), four were discarded because the quality of the image, as seen on the computer screen, was considered unsatisfactory. In considering the group consisted of all raters, there were 651 agreements and 385 disagreements.

According to the World Health Organization (WHO),^14^ the correlation between raters after calibration, particularly in evaluating oral conditions,should reach values ranging from 85% to 95%. Given that the subjective diagnosis of facial patterns is recent and not yet fully established, a minimum percentage of 70% was regarded as acceptable in terms of accuracy. In other words, 30% of error was set as the limit to consider the method’s results satisfactory. Rater agreement with the gold standard reached 62.8%. As it can be observed in Table 6, the experienced group’s accuracy was 66.4%, thus almost reaching the critical value (70%), while the inexperienced group’s accuracy was 58.2%.These results allow one to argue that the subjective criteria guiding the classification of facial patterns improve as professionals mature.

It should be noted that accuracy observed in the group of experienced examiners represents the maximum accuracy of the method, as this group consisted of orthodontic professors selected by the method’s author who were also recognized for having great familiarity with such diagnostic system. Therefore, in order to assess the method’s average result, the results of all examiners were included in the calculation of the operational characteristics of each facial pattern (Table 7).

Evaluation of diagnostic tests usually investigates both the individual’s chances of developing the disease due to positive results and the chances of not developing the disease due to negative results. Such approach predominates in dichotomous tests in which the individual is classified only into two types: healthy or unhealthy. In this study, facial morphology classification comprised five options, among which only Pattern I attests to the morphological balance of the face. As a result, in ruling out Patterns II, III, Long Face or Short Face in a given individual, it would not be possible to argue that such individual has a balanced facial pattern, since he or she might belong to any of the four remaining facial classification alternatives. Given the polytomous nature of facial pattern classification, the results pertaining to the chances of an individual not having a particular facial pattern were left out. Therefore, the investigation into the application of the subjective method of facial pattern classification is focused on answering two questions:

1. What is the method’s ability to identify which individual belongs to each pattern? (Sensitivity).
2. What are the chances of a facial pattern assigned to an individual being correct? (Predictive value).

A minimum percentage of 70% was adopted for both sensitivity and positive predictive value. Therefore, in order to be considered an adequate performance, the test would have to reach values ​​above 70% in both evaluations. On the issue of sensitivity, the method performed satisfactorily in the Short Face pattern (91.6%) and unsatisfactory in Patterns I, II, III and the Long Face pattern; whereas positive predictive value proved satisfactory in Pattern II (79.9%), and inadequate in Patterns I, III, as well as Long Face and Short Face patterns (Table 7).

One limitation of the positive predictive value lies in its dependence on the proportion of “unhealthy” subjects in the sample.^15^ For that reason, it was decided to complement the assessment of the subjective method of facial pattern classification by means of the positive likelihood ratio, which expresses how many times more likely it is for a positive test result to occur in healthy versus unhealthy subjects. The likelihood ratio showed high values ​​for all patterns, except for Pattern I.

In the diagnostic field, the likelihood ratio itself does not evaluate test performance; clinical efficiency is expressed by post-test probability, which depends on the relationship between the likelihood ratio and clinical prevalence.^15^ To calculate post-test probability, the author used the estimated clinical prevalence of facial patterns based on quotes gleaned from the book of Capelozza Filho.^5^ It can be observed in Figure 8 that even with high positive likelihood ratios, the post-test probability in Pattern III, Long Face and Short Face patterns proved inadequate. Apparently, in cases in which prevalence is low, the efficacy of a diagnostic method requires extreme ability to identify unhealthy individuals, and a minimum percentage of confounders between patterns.

The confounding factors in each pattern can be seen in Table 8. In Patterns II and III, approximately half of confounders were related to vertical patterns and the other half was related to Pattern I; that is, the morphological criteria proved effective in discriminating opposite sagittal patterns (II and III). However, bordering patterns (I/II and I/III) need improvement. Likewise, the morphologic criteria were effective to distinguish Short Face and Long Face patterns, since 100% of discrepancies with regard to the gold standard in vertical patterns involved confounding variables with sagittal patterns. It was found, therefore, that most of the errors in classifying facial patterns occurred in complementary direction, i.e., the gold standard provided the classification in the sagittal direction, while the rater provided it in the vertical direction, or vice-versa.

These results prompted two questions:

1) Should disagreements in different space planes be considered pattern classification errors? No, given that often deviations in the sagittal transverse and vertical components are associated in dentoskeletal soft tissue imbalance. Moyers et al^2^ stresses this concept in his basic morphological analysis which proposes the integrated diagnosis of vertical and sagittal skeletal components in a non-exclusionary approach.
2) The current method configuration exhibits a character of integration or opposition between the vertical and sagittal vectors? To answer this question, one must bear in mind that the diagnostic system proposed by Capelozza Filho^5^ already recognizes the joint participation of vertical and sagittal vectors. However, in defining the facial pattern, this method formally classifies only the vector that displays the greatest morphological deviation, providing the basis for a facial diagnosis, while the complementary vector is included informally. Although the implicit goal of classifying sagittal and vertical vectors undoubtedly exists, apparently, the current configuration discloses in practice an exclusive feature of this method to the extent that it allows a selective diagnostic classification between vertical and/or sagittal vectors. Therefore, it is suggested that a two-factor evaluation be formally established:

a) Classification of the primary vector responsible for the facial pattern.
b) Classification of the associated complementary vector.

One last confounding factor may be related to an ethnic factor, given that in its core the method was based on the characteristics of Caucasian individuals. Today research is aiming to establish the morphological features of Pattern I in Asians and African-Americans.

 This study allowed an overview of the subjective method of facial classification; however, future research is recommended to correct some methodological limitations identified in this study, such as the number of examiners and sample size, which should be increased, especially in the Short Face group, so as to avoid Type II error. Finally, since the orthodontists were not randomly selected from a larger pool, results cannot be generalized to all practicing orthodontists.

### Concluding remarks

According to Vieira and Hossne,^16^ if in a given experiment the groups being compared are distinguished only by the sort of treatment, it is logical to infer that treatment is the cause of difference between groups. However, if groups differ with regard to factors other than treatment, differences between them can be wholly or partly due to these other factors; i.e., confounders between treatment and other factors. In this context, differential diagnosis of facial patterns is an essential goal in Orthodontics to avoid confusion caused by different patterns of craniofacial growth. Although the results of this study indicate the need for improvement in the morphological criteria defining facial patterns, it is a worthwhile method to the extent that it contains a diagnostic system in line with the aspirations of scientific research, as it distinguishes among individuals with similar morphological aspects. By doing so, the procedure plays a pivotal role in furthering the practice of evidence-based Orthodontics and Facial Orthopedics.

## CONCLUSIONS

Within the limitations of the present study, the objective was to investigate the performance of the subjective method of facial pattern classification when applied to adults. However, because it involves the classification of five facial patterns, operating results were not uniform. Therefore, the conclusions derived from the method were divided into three levels of performance:

» Satisfactory performance: reproducibility, sensitivity in the Short Face pattern and predictive value in Patterns I and II.
» Slightly below satisfactory performance: sensitivity in Patterns I, II, III and Long Face, as well as predictive value in the Long Face. Because performance showed results that are close to the minimum acceptable value in this investigation, the stringency with which examiners are trained and calibrated should be further increased and performance tests repeated.
» Unsatisfactory performance: predictive value in Patterns III and Long Face. Due to the low clinical prevalence of such patterns, it is suggested that discriminating morphological criteria be improved.
